# Is diabetes associated with malaria and malaria severity? A systematic review of observational studies

**DOI:** 10.12688/wellcomeopenres.15467.3

**Published:** 2019-12-11

**Authors:** Rodrigo M. Carrillo-Larco, Carlos Altez-Fernandez, Cesar Ugarte-Gil

**Affiliations:** 1 Department of Epidemiology and Biostatistics, School of Public Health, Imperial College London, London, UK; 2 CRONICAS Centre of Excellence in Chronic Diseases, Universidad Peruana Cayetano Heredia, Lima, Peru; 3 Centro de Estudios de Poblacion, Universidad Catolica los Ángeles de Chimbote (ULADECH Catolica), Chimbote, Peru; 4 Facultad de Medicina Alberto Hurtado, Universidad Peruana Cayetano Heredia, Lima, Peru; 5 Instituto de Medicina Tropical Alexander von Humboldt, Universidad Peruana Cayetano Heredia, Lima, Peru; 6 Department of Clinical Research, London School of Hygiene and Tropical Medicine, London, UK; 7 Department of International Health, Johns Hopkins Bloomberg School of Public Health, Baltimore, USA

**Keywords:** malaria, diabetes, multi-morbidity, co-morbidity, syndemics, Peru

## Abstract

**Background: **We conducted a systematic review to study the association between diabetes and malaria as well as malaria severity.

**Methods: **The search was conducted in Embase, Global Health, MEDLINE, Scopus and Web of Science. Titles and abstracts were screened, full-text studied and information extracted for qualitative synthesis. Risk of bias was assessed with ROBINS-I criteria. The exposure was diabetes and the outcome malaria or malaria severity.

**Results: **Of 1992 results, three studies were included (n=7,226). Two studies found strong associations: people with diabetes had higher odds of malaria (adjusted odds ratio (aOR): 1.46 (95% CI: 1.06-2.03)) and severe malaria (aOR: 2.98 (95% CI: 1.25-7.09)). One study did not find conclusive evidence: aOR for severe malaria was 0.95 (95% CI: 0.71-1.28). Risk of bias was high in all the studies.

**Conclusions: **Although the available evidence on the association between diabetes and malaria is limited, the results may suggest there is a non-trivial positive relationship between these conditions.

## Introduction

In a global context where non-communicable diseases lead the ranking of mortality and disease burden, malaria is a tropical disease still accountable for thousands of years of life lost and years lived with disability disproportionally affecting low- and middle-income countries (LMICs) ^1, 2^. The current multi-morbidity and syndemics paradigm calls to study diseases as clusters rather than isolate entities ^3, 4^, and new links between diseases could signal innovative prevention paths, e.g., adequate control of socio-demographic determinants for both conditions. Moreover, this knowledge could also provide relevant evidence on treatment and management, e.g., medications and their interactions for subjects with both illnesses. Therefore, looking at unknown associations between non-communicable diseases and malaria seems relevant, particularly for diseases for which incidence has swiftly risen in the last decades such as diabetes ^5– 7^. Even though there is evidence about the increasing co-morbidity between diabetes and infectious diseases ^7, 8^, the concept that diabetes could be a “risk factor” for malaria is relatively new ^7, 9^. Although relevant, available evidence is still sparse, including letters, brief reviews and animal models ^10– 12^. To the best of our knowledge, no systematic review has yet summarised the evidence on the association between diabetes and malaria. In so doing, the magnitude of this association could be explored, and research gaps identified. Consequently, we aimed to conduct a systematic review of observational studies addressing the association between diabetes and malaria as well as the association between diabetes and malaria severity.

## Methods

### Study design

This is a systematic review of the literature following the PRISMA guidelines ^13^ *Extended data* (p. 2); the checklist is available on Figshare ^14^. This work was registered at PROPSERO (ID CRD42018105771).

### Search strategy

The search was conducted in Ovid, including Embase, Global Health and MEDLINE, as well as in Scopus and Web of Science; for specific terms used in these search engines please refer to *Extended data* (pp. 4–7). The search was conducted from inception to July 31 ^st^ 2018, and no language restrictions were set. However, the search in Ovid was restricted to human subjects; the search in Scopus was restricted to articles and medicine as subject area; and the search in Web of Science was restricted to articles.

### Selection criteria

We sought studies that included humans subjects, had a comparator group (e.g., healthy individuals or subjects without diabetes), and the outcome was malaria diagnosis or severity. In detail, studies were selected if they included human subjects regardless of where they had been enrolled, i.e., these could have been population-based/community studies or hospital-based samples; no age restrictions were set. The study followed an observational design, e.g., cross-sectional, case-control or cohort study. Case reports and non-comparative studies were excluded. The exposure variable was diabetes defined as either a laboratory test (e.g., fasting plasma glucose ≥126 mg/dL), self-reported diagnosis or currently receiving medication for diabetes; these variables could have been actively collected or retrieved from medical records. The exposure of interest could have been any kind of diabetes (e.g., type 1 or type 2). The outcome was either malaria diagnosis or malaria severity regardless of the species as defined in each original report; for example, cases could should have had laboratory confirmation (e.g., blood smears, rapid diagnostic tests or polymerase chain reactions (PCR)) or based on clinical or discharge records.

### Data collection

Using the Rayyan online tool ^15^, two reviewers (RMC-L and CA-F) independently screened titles and abstracts retrieved from the search strategy (agreement 0.99 and Kappa 0.99, *Extended data (p. 8)*). The full text of the studies that both reviewers agreed met selection criteria were sought; in addition, studies on which both reviewers had a discrepant opinion were also retrieved in full text. These full texts were studied by two reviewers (RMC-L and CA-F) independently to select those for final inclusion. The authors agreed on items that needed to be extracted from each study and RMC-L sought these pieces of information; all the authors reviewed the extraction process.

Risk of bias was assessed using the ROBINS-I criteria ^16^; nevertheless, the “Bias due to deviations from intended interventions” criterion was not considered because it did not apply to the studies herein included. RMC-L conducted the risk of bias assessment and all the authors reviewed the results.

The results are presented as a qualitative synthesis. No meta-analysis was planned because very few studies were expected, with high variability among them. Therefore, summary estimates (e.g., odds ratio) are summarized and presented as they were reported by each original study.

## Results

### Search strategy

**Figure 1.**
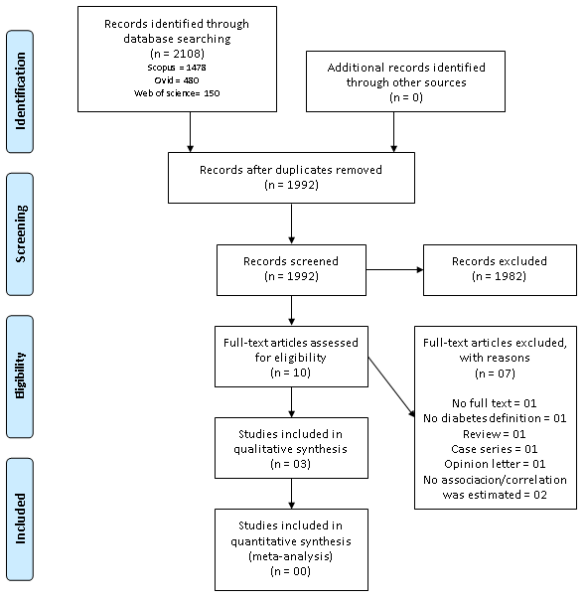
Flow chart of the study selection process.

The search strategy retrieved 2108 results, 1992 were screened and 10 selected for in-depth scrutiny; finally, three studies were included for qualitative synthesis. Figure 1 depicts the number of studies at each stage of the screening process.

### Study characteristics

Table 1 shows the overall characteristics of the selected studies. Of the three studies, two were conducted in high-income countries: USA ^17^ and Sweden ^18^; whereas one was in Africa (Ghana) ^19^. All of them were published in the last 8 years and followed a retrospective design. There was heterogeneity in diabetes definition, including blood tests (e.g., fasting glucose) and history based on clinical records.

**Table 1.** Characteristic of the studies included in the systematic review and reported measures of association.

Study	Country	Design	Setting	Study population	Sample size	% diabetes/ % malaria	Exposure	Outcome	Main result
Malaria Susceptibility									
Danquah I, *et al.* ^19^ (2010)	Ghana	Case-control study	Health centre	Men and women aged ≥18 years	1,466	46% / by PCR 14.1% had *Plasmodium spp.* infection (91.8% *P. falciparum*)	Type 2 diabetes: treatment or fasting plasma glucose ≥7 mmol/L	Malaria: PCR was used to identify *Plasmodium* infection and species	Type 2 diabetes was associated with higher odds of *Plasmodium* *falciparum* infection: aOR = 1.46 (95% CI: 1.06-2.03)
Malaria Severity									
Wyss K, *et al.* ^18^ (2017)	Sweden	Retrospective observational study	National surveillance data (Public Health Agency)	All adults (≥18 years) with *Plasmodium* *falciparum* microbiologically confirmed	937 individuals with *P. falciparum*, of which 9.8% were severe	In severe cases 9.8% had diabetes	Diabetes: as per the ICD-10 codes registered, including type 1, type 2 and unspecified	Severe malaria: 2012 WHO criteria (modified by the authors)	Regardless of hyperparasitemia, diabetes was associated with higher odds of severe malaria: aOR = 2.98 (95% CI: 1.25-7.09)
Khuu D, *et al.* ^17^ (2018)	USA	Retrospective observational study	Based on hospital records from State Inpatient Database	Men and women whose discharge records had malaria (ICD-9 codes) as the primary or secondary diagnoses	4,823 severe malaria hospitalizations	Among severe malaria inpatients, 10.4% type 2 diabetes	Based on the hospital records; no further details	Severe malaria: CDC criteria (modified by the authors). Malaria complications: where the discharge record noted malaria plus one or more complications (e.g., neurological symptoms)	Type 2 diabetes was not associated with severe malaria or malaria plus complications: aOR = 0.95 (95% CI: 0.71- 1.28) [severe malaria]; aOR = 1.06 (95% CI: 0.60-1.88) [malaria with ARDS]; aOR = 0.93 (95% CI: 0.55-1.57) [cerebral malaria]; aOR = 1.16 (95% CI: 0.75-1.80) [malaria with severe anaemia]; aOR = 1.20 (95% CI: 0.82-1.74) [malaria with renal failure]; aOR = 0.83 (95% CI: 0.36-1.92) [malaria with jaundice]

### Diabetes and malaria susceptibility

One of the three selected reports showed a strong positive association between diabetes and malaria susceptibility. People with type 2 diabetes had higher odds of malaria: adjusted odds ratios (aOR) = 1.46; 95% CI: 1.06-2.03 ( Table 1) ^19^.

### Diabetes and malaria severity

Of the three selected reports, one provided strong evidence that people with diabetes (including type 1 and 2) had higher odds of severe malaria, aOR = 2.98; 95% CI: 1.25-7.09 ^18^. One study did not find a strong association, reporting that type 2 diabetes and severe malaria had an aOR = 0.95; 95% CI: 0.71-1.28 ( Table 1) ^17^.

### Risk of bias

The risk of bias assessment showed that the three retrieved studies had critical risk of bias. They all had low bias in classification of the intervention, and they presented poor information to assess bias due to missing data. Conversely, the report by Wyss and colleagues was deemed as low risk of bias in the participant selection domain ^18^. Table 2 summarizes all criteria for risk of bias assessment, and specific items within each criterion are shown in *Extended data* (pp. 9–11) ^14^.

**Table 2.** Risk of bias assessment.

Variable	Study		
	Danquah I, *et al.* ^19^	Wyss K, *et al.* ^18^	Khuu D, *et al.* ^17^
Bias due to confounding	Serious risk of bias	Serious risk of bias	Serious risk of bias
Bias in selection of participants into the study	Critical risk of bias	Low risk of bias	Critical risk of bias
Bias in classification of interventions	Low risk of bias	Low risk of bias	Low risk of bias
Bias due to missing data	No information	No information	No information
Bias in measurement of outcomes	Moderate risk of bias	Moderate risk of bias	Moderate risk of bias
Bias in selection of the reported result	Moderate risk of bias	Moderate risk of bias	Moderate risk of bias
**Judgement**	**Critical risk of bias**	**Critical risk of bias**	**Critical risk of bias**

## Discussion

### Summary of evidence

There were three reports addressing the association between diabetes and malaria, one studied malaria diagnosis ^19^ whereas two malaria severity or complications ^17, 18^. There was a strong positive association between diabetes and malaria diagnosis ^19^, and only one study found compelling evidence of an association between diabetes and malaria severity ^18^. Across these three studies, overall risk of bias was high.

### Limitations

The study designs followed by the reviewed studies were case-control ^19^ and retrospective analyses ^17, 18^. Although these methodologies provide relevant evidence and have several strengths, regarding the topic at hand the evidence they offer still needs further verification with stronger observational approaches. These could include prospective cohorts or thoroughly conceived analysis following a casual-inference methodology. On the other hand, we acknowledge that the reviewed studies ascertained both the exposure and outcome following valid methodologies, which in turn suggests that addressing the association of interest is feasible and supports our call for further studies in this field.

Differences in malaria severity definition could limit our conclusions too. One report ascertained malaria severity with laboratory tests ^18^, and the other extracted the information from hospital discharge records ^17^. The second report was included in the review because hospital records are most likely to be based on laboratory tests, even though this was not disclosed in the publication. The different methodologies these studies followed could explain why the former report showed a strong association whereas the latter failed to show compelling evidence, even though they both had a similar proportion of people with diabetes among severe malaria cases ^17, 18^.

This review has some strengths and limitations. First, we searched five international search engines, which allowed us to cover thousands of published materials. Therefore, our results show what there is available at a global scale and thus signal a dearth of strong evidence on the association between diabetes and malaria. Second, there are several tools to assess risk of bias. The one we followed, ROBINS-I, is a domain-based approach which seems to be a better approach in comparison to tools without domains; nevertheless, it has also been suggested that ROBINS-I labels more studies as of high risk of bias ^20^. Regardless of the assessed domains and other properties of the ROBINS-I, the study design (e.g., case-control) followed by the reviewed reports already suggest high risk of bias. This is not a critique on the available evidence, but rather a call to build on the available literature and conduct more comprehensive research. Third, we defined malaria and malaria severity as in the original publications. Differences in malaria definitions could explain some results as discussed above ^17, 18^. Nonetheless, two of the three selected reports confirmed their cases with laboratory methods while one used discharge records. The positive and strong association these two publications found ^18, 19^, supports the potential association between diabetes and malaria, while also signalling the dearth of evidence on this subject.

### Implications for research

This systematic review found three studies following a case-control ^19^ and retrospective analysis approach ^17, 18^. Although they did a great effort to ascertain the exposure and outcome with precision, e.g., using biomarkers for diabetes or PCR for malaria, they lacked generalizability because their study populations were based on (small) patients samples instead of general population samples. The selected reports also followed the most suitable research design given their locations; for example, a case-control study was feasible for Danquah and colleagues in Ghana where malaria is prevalent ^19^, though it would be challenging for other researchers in USA and Sweden where retrospective analysis based on clinical records is much efficient ^17, 18^. All these studies have provided relevant evidence and have sparked interest in this novel yet relevant study field: diabetes and malaria. 

 A relevant research approach, as suggested by Broz *et al.*, would be how to improve diabetes control in people with malaria ^21^. Because malaria would imply haemolysis, this could impair the accuracy of HbA1c to inform about diabetes control; potential solutions could include daily glucose monitor or fructosamine ^22^. We believe that a population-based cohort study that has assessed diabetes would be a key asset in further elucidating the (true) association between diabetes and malaria. We are confident that these studies are available somewhere, with interest in LMICs, and that soon they will produce evidence in order to identify whether addressing diabetes could reduce malaria burden.

### Implications for public health and clinical practice

At this this time we believe it is premature to draw any strong conclusions for clinical practice or public health. Nevertheless, we could suggest strengthening malaria prevention strategies for people with diabetes, particularly in highly endemic areas or among travellers to these settings. In accordance to international guidelines suggesting prophylactic treatment for travels to malaria highly-endemic areas ^23, 24^, these recommendations could be stronger for people with diabetes.

### Diabetes and malaria: the epidemiological context

Global estimates inform that Africa is the region with the largest number of Malaria cases, where Nigeria, followed by the Democratic Republic of Congo, accounts for most of these ^25^. Although there has been a decrease since 1990, West and Central Africa exhibit the highest malaria death rates, with Mali, Burkina Faso and Niger leading the ranking ^26^. On the other hand, epidemiological evidence shows that from 1980 to 2014, diabetes prevalence has doubled in most sub-Saharan nations ^5, 6^. Regarding the above highlighted countries, diabetes prevalence in Nigeria and the Democratic Republic of Congo has increased by 2- and 2.5-fold, respectively; these estimates for Mali, Burkina Faso and Niger were 2.6-, 2.8- and 2.3-fold ^5, 6^. These country-level estimates could have several potential implications. First, countries with high malaria prevalence have experienced a dramatic increase in diabetes prevalence. Perhaps, this could imply that the rising diabetes burden makes it difficult to lessen malaria burden despite large efforts on this matter. Second, Burkina Faso, one of the countries with high malaria death rates have also had an utterly increase in diabetes prevalence. This could suggest that the increasing diabetes burden may be leading to more severe cases with fatal outcomes. Third, as diabetes prevalence keeps rising in malaria endemic areas, larger populations could be at high risk of malaria. This could lead to call to strengthen diabetes prevention and early diagnosis programs, not only to stop non-communicable diseases but also because of a potential positive impact on malaria control. These ecological arguments, along with the evidence summarized in this review, suggest of a possible co-morbidity (if not synergism) between diabetes and malaria that deserves further and comprehensive scrutiny.

### Diabetes and malaria: pathways

The aim of this review was to synthetize the evidence about diabetes as an associated factor with higher malaria prevalence, incidence or severity. Even though we could not assess diabetes as a ‘risk factor’ in the strict definition of the term because of the lack of prospective studies, we summarized preliminary evidence that suggest diabetes could be associated with malaria and malaria severity. In addition to this finding, biological pathways have been proposed to explain this association.

Olivier and colleagues summarized key aspects of immune response in malaria, highlighting features of early infection including that phagocytes such as monocytes, macrophages and neutrophils lead the immunological response ^27^; this role of monocytes and macrophages during erythrocyte infection has been studied before, particularly in response to *Plasmodium* hemozoin and Glycosylphosphatidylinositol ^28– 31^. However, macrophage phagocytic activity seems to be reduced in diabetics ^32– 35^, and this is paramount in the association between diabetes and tuberculosis ^36^. This impaired immunity, particularly regarding those cells with responsibilities against malaria, could explain the association between diabetes and malaria ^11^. These arguments, i.e. impaired immunity, support solid hypotheses to explain higher malaria susceptibility and malaria severity among people with diabetes.

Other possible pathways between diabetes and malaria include hyperinsulinemia, which has been suggested to affect mosquitos in a way that they have a reduced immune response against *Plasmodium falciparum* ^37^. This implies there would be more mosquitos to spread malaria. Although we are unaware of robust evidence to support this potential explanation, Raghunath hypothesised that diabetics who take metformin would attract more mosquitos because this medication increases the concentration of lactic acid in their sweat, a compound that attracts mosquitoes ^10^; however, biguanides appear to positively interact with anti-malaria drugs ^38^. These additional arguments to support the association between diabetes and malaria render further importance to the study of this co-morbidity; not only because this would enrich science, but because it could also provide innovative prevention or treatment strategies.

## Conclusions

Despite conducting a comprehensive systematic review, the results showed there is paucity in the evidence about the association between diabetes and malaria as well as between diabetes and malaria severity. Of the three reviewed studies, one found a strong association between diabetes and malaria diagnosis, and one between diabetes and malaria severity; one study did not find strong evidence of association between diabetes and malaria severity. The three studies had high risk of bias, with one following a case-control design while the other two conducted a retrospective analysis. This limited evidence precludes drawing a strong conclusion on the associations of interest. Although there is evidence signalling biologically plausible pathways between diabetes and malaria as well as malaria severity. It is still premature to make clinical or public health recommendations to address the (possible) synergism between diabetes and malaria. On the other hand, this review signals the dearth of evidence on this association, both in terms of quantity and quality of available research; this calls to further study these associations and others between communicable and non-communicable diseases particularly in places with high or raising burden of both.

## Data availability

### Underlying data

All data underlying the results are available as part of the article and no additional source data are required.

### Extended data

Figshare: IS DIABETES ASSOCIATED WITH MALARIA AND MALARIA SEVERITY? A SYSTEMATIC REVIEW OF OBSERVATIONAL STUDIES - SUPPLEMENTARY MATERIAL. https://doi.org/10.6084/m9.figshare.9789740.v2 ^14^.

### Reporting guidelines

Figshare: PRISMA checklist for ‘Is diabetes associated with malaria and malaria severity? A systematic review of observational studies’. https://doi.org/10.6084/m9.figshare.9789740.v1 ^14^.

Data are available under the terms of the Creative Commons Attribution 4.0 International license (CC-BY 4.0).
