# Supplementary material for: Is diabetes associated with malaria and malaria severity? A systematic review of observational studies
Source: Wellcome Open Res. 2019 Dec 11;4:136. Originally published 2019 Sep 19. [Version 3] doi: 10.12688/wellcomeopenres.15467.3 (PMC6961413; doi:10.12688/wellcomeopenres.15467.3)
Supplement: Peer review history

# Peer review: Reviewer response for version 3

**DOI:** 10.21956/wellcomeopenres.17124.r37372

**Published:** 2020-01-13

**Authors:** Katja Wyss

**Affiliations:** 1 Division of Infectious Diseases, Department of Medicine Solna, Karolinska Institute, Stockholm, Sweden

I confirm that I have read this submission and believe that I have an appropriate level of expertise to confirm that it is of an acceptable scientific standard.

# Peer review: Reviewer response for version 2

**DOI:** 10.21956/wellcomeopenres.17091.r37148

**Published:** 2019-12-02

**Authors:** Katja Wyss

**Affiliations:** 1 Division of Infectious Diseases, Department of Medicine Solna, Karolinska Institute, Stockholm, Sweden

I think the authors adequately have addressed my questions and revised the manuscript accordingly except for one aspect: Concerning the definition of malaria, the authors state that “because the work by Khuu and colleagues ^1^ was based on hospital discharge records from a high-income country, we strongly believe these cases were based on laboratory tests hence the inclusion on the review. In fact, because they based the cases selection on ICD codes, these should have been based on laboratory tests to confirm the diagnostics.” I do not object to the inclusion of the study by Khuu et al, however I strongly advocate the authors to correct the selection criteria in the method section. A malaria ICD code cannot be assumed to be the same as microbiological confirmed malaria, discharge records usually include several discharge diagnosis and these constitute the basis for financial compensation in many high-income countries, thus could also include for example previous malaria in the medical history of the patient or an investigation for suspected malaria. Khuu et al have also discussed this in the limitation section of their paper. Also of importance; if the authors have excluded any studies using malaria ICD codes instead of microbiological confirmed malaria it would not be correct to revise the selection criteria but instead specify that an exception was done for the study by Khuu et al.

I confirm that I have read this submission and believe that I have an appropriate level of expertise to confirm that it is of an acceptable scientific standard, however I have significant reservations, as outlined above.

## References

[1] : Risk factors for severe malaria among hospitalized patients in the United States, 2000–2014. *Infection, Disease & Health*.2018;23(2) : 10.1016/j.idh.2018.01.002 93-106 10.1016/j.idh.2018.01.002 38715309

# Response

**Published:** 2019-12-02

**Authors:** Rodrigo Carrillo-Larco

**Affiliations:** Imperial College London, UK

We have modified the selection criteria section as well as the limitations of the study. The new lines read, respectively:

*The outcome was either malaria diagnosis or malaria severity regardless of the species as defined in each original report; for example, cases could have had laboratory confirmation (e.g., blood smears, rapid diagnostic tests or polymerase chain reactions (PCR)) or based on clinical or discharge records.*

*Third, we defined malaria and malaria severity as in the original publications. Differences in malaria definitions could explain some results as discussed above ^17, 18 ^. Nonetheless, two of the three selected reports confirmed their cases with laboratory methods while one used discharge records. The positive and strong association these two publications found, ^18, 19^ supports the potential association between diabetes and malaria, while also signalling the dearth of evidence on this subject.*

# Peer review: Reviewer response for version 2

**DOI:** 10.21956/wellcomeopenres.17091.r37149

**Published:** 2019-11-26

**Authors:** Raghunath Pendru

**Affiliations:** 1 Faculty of Medicine, Department of Microbiology, Texila American University, Georgetown, Guyana

The authors have revised the manuscript according to reviewers' suggestion. The revised manuscript is significantly improved and  I feel that this manuscript is suitable for indexing in the current form.

I confirm that I have read this submission and believe that I have an appropriate level of expertise to confirm that it is of an acceptable scientific standard.

# Peer review: Reviewer response for version 1

**DOI:** 10.21956/wellcomeopenres.16914.r36961

**Published:** 2019-11-18

**Authors:** Raghunath Pendru

**Affiliations:** 1 Faculty of Medicine, Department of Microbiology, Texila American University, Georgetown, Guyana

This study is a systematic review to find an association between diabetes and Malaria. Study is highly relevant because diabetes patients are susceptible to various infections because of dysregulation of immune response. Authors have finally selected only three articles addressing the association between diabetes and malaria. One of those articles focused on malaria diagnosis and two others studied malaria severity or complications. Only one study found compelling evidence of an association between diabetes and malaria severity. Authors need to clarify why they have finally selected only three studies for qualitative synthesis from 1992 studies. Ten articles that were selected for in-depth scrutiny should be considered for synthesizing the data. I recommend that they should also include those seven full-text articles for analysis and submit the results as supplementary materials.

 Though it is a systemic review, after analysis of the selected articles, authors should be able to form good hypotheses to explain the reasons for high susceptibility of diabetics to malaria infection. They should also be able to explain the reasons for high severity and complications of malaria in diabetics. This study is well designed and deserves indexing after incorporating results from more studies.

I confirm that I have read this submission and believe that I have an appropriate level of expertise to confirm that it is of an acceptable scientific standard, however I have significant reservations, as outlined above.

# Response

**Published:** 2019-11-19

**Authors:** Rodrigo Carrillo-Larco

**Affiliations:** Imperial College London, UK

**Q1: Authors need to clarify why they have finally selected only three studies for qualitative synthesis from 1992 studies. Ten articles that were selected for in-depth scrutiny should be considered for synthesizing the data. I recommend that they should also include those seven full-text articles for analysis and submit the results as supplementary materials.**

A1: The three studies finally included in the review were those which met the inclusion criteria detailed in the methodology. Of the ten studies that were selected after the titles/abstract screening, seven were excluded because these did not meet the inclusion criteria. To be consistent with our methodology, and inclusion/exclusion criteria as in any thorough systematic review, we cannot include more results in the review because these do not answer the research question. However, as suggested, we have listed the seven excluded reports in supplementary material (table below). 

BORN WC. [Malaria and diabetes mellitus]. Z Tropenmed Parasitol. 1952;3(4):503-7. Undetermined Language. PubMed PMID: 14959277.

Alicke M, Boakye-Appiah JK, Abdul-Jalil I, Henze A, van der Giet M, Schulze MB, Schweigert FJ, Mockenhaupt FP, Bedu-Addo G, Danquah I. Adolescent health in rural Ghana: A cross-sectional study on the co-occurrence of infectious diseases, malnutrition and cardio-metabolic risk factors. PLoS One. 2017;12(7):e0180436. doi: 10.1371/journal.pone.0180436.

Adeleke SI, Asani MO, Belonwu RO, Gwarzo GD, Farouk ZL. Childhood diabetes mellitus in Kano, North West, Nigeria. Niger J Med. 2010;19(2):145-7.

Raghunath P. Impact of type 2 diabetes mellitus on the incidence of malaria. J Infect Public Health. 2017;10(3):357-358.

Ahmed AM, Elawad AM, Saeed OK. Malaria: a question of an etiological role in diabetes mellitus. East Mediterr Health J. 1999;5(4):821-2.

Kalra S, Khandelwal D, Singla R, Aggarwal S, Dutta D. Malaria and diabetes. J Pak Med Assoc. 2017;67(5):810-813.

Mohapatra, Manoj. (2001). Profile of severe falciparum malaria in diabetics. International Journal of Diabetes in Developing Countries. 21.

**Q2: Though it is a systemic review, after analysis of the selected articles, authors should be able to form good hypotheses to explain the reasons for high susceptibility of diabetics to malaria infection. They should also be able to explain the reasons for high severity and complications of malaria in diabetics.**

A2: We have made it clear what pathophysiological hypothesis could explain the associations of interest (page 10 updated manuscript).

# Peer review: Reviewer response for version 1

**DOI:** 10.21956/wellcomeopenres.16914.r36656

**Published:** 2019-11-05

**Authors:** Katja Wyss

**Affiliations:** 1 Division of Infectious Diseases, Department of Medicine Solna, Karolinska Institute, Stockholm, Sweden

This systematic review investigated if there is an association between diabetes and malaria as well as diabetes and severity of malaria. The objectives of the review are highly relevant considering the rapid increase in non-communicable diseases like diabetes in countries that at the same time are carrying a heavy malaria burden. However, although a comprehensive literature search was performed, only three studies fulfilled inclusion criteria, and the review has some additional important limitations that need to be addressed.

Abstract/Introduction:

 It should be emphasised, as the authors have done in the title but not in the background of neither abstract nor main text, that the aim of the review includes two completely different research questions: 1) If diabetes affects malaria susceptibility and 2) If diabetes affects severity of malaria.

 The sentence in the background section of the abstract needs to be rephrased: “We conducted a systematic review to study the association between diabetes as a risk factor for malaria”, change to either *We conducted a systematic review to study the association between diabetes and … *or to *We conducted a systematic review to study if diabetes is a risk factor for…*

Methods:

 Selection criteria: Concerning definition of outcome the authors need to specify how severe malaria was defined and revise the definition of malaria cases as laboratory confirmed, see below under results.

 Data collection: Suggest that the authors specify in the methods section if screening of articles were done independently by the two reviewers and also include the proportion of agreement between reviewers in the screening process (not only as supplement).

Results:

 Ten articles were selected and assessed for eligibility, it would be recommended to add (as supplementary materials) a list of the seven full-text articles that were excluded.

 Study characteristics: The authors state that “In all the studies the malaria diagnosis was confirmed with blood tests, including PCR.” This is however not correct; the malaria cases in the study by Khuu et al were identified by ICD-9 discharge diagnoses since laboratory results were not included in the NIS (Nationwide Inpatient Sample). This is an important difference and limits the comparison with the study by Wyss et al. In addition, this implies either that the authors need to exclude this study since the case definition does not fit with the one specified under methods, or they need to redo the literature search using a broader definition of malaria, including any additional studies with parasitological un-confirmed cases.

 Diabetes and malaria susceptibility: It is important to point out to the readers that the increased risk in this group of diabetics concerned asymptomatic parasitaemia, none of the included subjects presented with fever.

 Diabetes and malaria severity: The sentence “Of the selected reports, two provided evidence that people with diabetes had higher odds of severe malaria” should be rephrased. Only one of the papers presented a significantly higher odds of severe malaria for diabetics. However, one could mention that in both studies there was a higher proportion of diabetics among severe cases compared to non-severe. Also, it is important to point out that assessment of severe malaria was based on different methods in the two retrospective studies.

 Risk of bias: I do not quite agree concerning the assessment of bias. Could the authors explain why there would be a selection bias in the study by Wyss *et al*. where all malaria cases reported to the national surveillance system were included? In contrast, including only hospital treated malaria patients could result in a selection bias. Also, concerning missing values, there is information about how missing values are handled in relevant tables and discussion for both the study by Khuu *et al*. and Wyss *et al*.

Discussion:

 Considering that only three articles were reviewed, it would be appropriate to provide a more thorough analysis concerning the diverging results of the two studies assessing risk factors for severe malaria. Any suggestions to why these studies showed different results? Both reported 10% diabetes among severe cases, however a lower proportion of diabetics among non-severe cases in the study by Wyss *et al*. The authors should consider if the difference could be associated with the selection of malaria cases and definition of severe malaria.

 Implications for research: Concerning generalizability, it is important to mention that the studies were done in different types of populations; while the cross-sectional studies were carried out in a non-endemic setting the case-control study was performed in a malaria endemic area where asymptomatic infections are common and severe manifestations of malaria seldom seen among immunocompetent, non-pregnant adults. Different study designs are required depending on setting and which research question one intends to answer, which also should be pointed out in the discussion. Using a general population sample of diabetics or a population based cohort study as the authors suggested would not be a reasonable approach in a non-endemic setting where the incidence of malaria is extremely low. In an endemic setting the challenge is instead lack of reliable health and recording systems as well as difficulty in the diagnostics of clinical malaria due to both frequent reinfections and asymptomatic parasitaemia.  

 Epidemiological context: As the authors emphasize, the prevalence of diabetes is increasing in many countries with high malaria transmission. However, I would suggest the authors to be a bit more careful in drawing conclusions concerning malaria mortality based on country estimates of diabetes and malaria prevalence.

 Pathways: The possible association of diabetes and risk of malaria infection, and risk of severe disease in an ongoing infection, implies completely different pathobiological mechanisms, which the authors should consider elaborating more on. As an example, there are several studies demonstrating that glucose levels affect parasite growth *in vitro,* which could have implications for malaria severity. Concerning the hypothesis that metformin use could attract mosquitos it would be reasonable to mention that the same drug also has antimalarial effect (Jones *et al *2002). ^1^

Conclusions:

 Again, important to clarify that there were two different objectives in this review, and the included studies analysed two distinct outcomes, thus it is not justified to simply conclude that there is an association between diabetes and malaria.

I confirm that I have read this submission and believe that I have an appropriate level of expertise to confirm that it is of an acceptable scientific standard, however I have significant reservations, as outlined above.

## References

[1] : Biguanide-atovaquone synergy against Plasmodium falciparum in vitro. *Antimicrob Agents Chemother*.2002;46(8) : 10.1128/aac.46.8.2700-2703.2002 2700-3 10.1128/aac.46.8.2700-2703.2002 12121961PMC127339

# Response

**Published:** 2019-11-19

**Authors:** Rodrigo Carrillo-Larco

**Affiliations:** Imperial College London, UK

**Reviewer #1**

**Q1: It should be emphasised, as the authors have done in the title but not in the background of neither abstract nor main text, that the aim of the review includes two completely different research questions: 1) If diabetes affects malaria susceptibility and 2) If diabetes affects severity of malaria. The sentence in the background section of the abstract needs to be rephrased: “We conducted a systematic review to study the association between diabetes as a risk factor for malaria”, change to either We conducted a systematic review to study the association between diabetes and … or to We conducted a systematic review to study if diabetes is a risk factor for…**

A1: The ‘background’ section of the abstract, as well as the last sentence of the introduction, have been modified as suggested to reflect that the review focused on both outcomes: malaria and malaria severity. 

**Q2: Selection criteria: Concerning definition of outcome the authors need to specify how severe malaria was defined and revise the definition of malaria cases as laboratory confirmed, see below under results.**

A2: Unlike malaria infection, for which we defined there should have been laboratory confirmation, we did not have a specific definition for malaria severity. We made the first decision to avoid bias or misclassification of malaria cases. However, we strongly believe that severe cases would be treated (and studied) in hospitals or other highly-specialized health facilities. Therefore, they are most likely to rely on laboratory tests to define severity, thus less likely to be subject of bias or misclassification. We have discussed these arguments in the ‘limitations’ section.

**Q3: Data collection: Suggest that the authors specify in the methods section if screening of articles were done independently by the two reviewers and also include the proportion of agreement between reviewers in the screening process (not only as supplement).**

A3: We have specified that both review stages (screening and full-text) were conducted by two reviewers independently. Also, as suggested, we have reported the agreement and Kappa metrics. Please refer to ‘data collection’ section for details.

**Q4: Ten articles were selected and assessed for eligibility, it would be recommended to add (as supplementary materials) a list of the seven full-text articles that were excluded.**

A4: We have included a list of the seven reports excluded at the second selection stage (full-text). The table below is in the updated supplementary material.

BORN WC. [Malaria and diabetes mellitus]. Z Tropenmed Parasitol. 1952;3(4):503-7. Undetermined Language. PubMed PMID: 14959277.

Alicke M, Boakye-Appiah JK, Abdul-Jalil I, Henze A, van der Giet M, Schulze MB, Schweigert FJ, Mockenhaupt FP, Bedu-Addo G, Danquah I. Adolescent health in rural Ghana: A cross-sectional study on the co-occurrence of infectious diseases, malnutrition and cardio-metabolic risk factors. PLoS One. 2017;12(7):e0180436. doi: 10.1371/journal.pone.0180436.

Adeleke SI, Asani MO, Belonwu RO, Gwarzo GD, Farouk ZL. Childhood diabetes mellitus in Kano, North West, Nigeria. Niger J Med. 2010;19(2):145-7.

Raghunath P. Impact of type 2 diabetes mellitus on the incidence of malaria. J Infect Public Health. 2017;10(3):357-358.

Ahmed AM, Elawad AM, Saeed OK. Malaria: a question of an etiological role in diabetes mellitus. East Mediterr Health J. 1999;5(4):821-2.

Kalra S, Khandelwal D, Singla R, Aggarwal S, Dutta D. Malaria and diabetes. J Pak Med Assoc. 2017;67(5):810-813.

Mohapatra, Manoj. (2001). Profile of severe falciparum malaria in diabetics. International Journal of Diabetes in Developing Countries. 21.

**Q5: Study characteristics: The authors state that “In all the studies the malaria diagnosis was confirmed with blood tests, including PCR.” This is however not correct; the malaria cases in the study by Khuu et al were identified by ICD-9 discharge diagnoses since laboratory results were not included in the NIS (Nationwide Inpatient Sample). This is an important difference and limits the comparison with the study by Wyss et al. In addition, this implies either that the authors need to exclude this study since the case definition does not fit with the one specified under methods, or they need to redo the literature search using a broader definition of malaria, including any additional studies with parasitological un-confirmed cases.**

A5: We were aware of the point highlighted by the reviewer, and we agree there may be differences that are now pinpointed in the ‘limitations’ section. Nevertheless, because the work by Khuu and colleagues was based on hospital discharge records from a high-income country, we strongly believe these cases were based on laboratory tests hence the inclusion on the review. In fact, because they based the cases selection on ICD codes, these should have been based on laboratory tests to confirm the diagnostic.

**Q6: Diabetes and malaria severity: The sentence “Of the selected reports, two provided evidence that people with diabetes had higher odds of severe malaria” should be rephrased. Only one of the papers presented a significantly higher odds of severe malaria for diabetics. However, one could mention that in both studies there was a higher proportion of diabetics among severe cases compared to non-severe. Also, it is important to point out that assessment of severe malaria was based on different methods in the two retrospective studies.**

A6: We have rephased the corresponding lines in the ‘results’ section to signal that only one study showed strong association. In addition, as pointed out before (Q5), the ‘limitations’ section now includes a few lines about the differences in ascertainment methods.

**Q7: Risk of bias: I do not quite agree concerning the assessment of bias. Could the authors explain why there would be a selection bias in the study by Wyss et al. where all malaria cases reported to the national surveillance system were included? In contrast, including only hospital treated malaria patients could result in a selection bias. Also, concerning missing values, there is information about how missing values are handled in relevant tables and discussion for both the study by Khuu et al. and Wyss et al.**

A7: We considered there was high risk of bias because these were cases reported to the national surveillance system. However, after further consideration and studying, we believe the surveillance system in Sweden should be good enough to register all malaria cases, especially severe cases as studied by Wyss and colleagues. Therefore, we re-classified this study as “low risk of bias” in the “Bias in selection of participants into the study” domain (also updated in supplementary material).

We considered “no information” on bias due to missing data because:

1. Wyss et al conducted multiple imputation for missing values in BMI: “To account for missing BMI, a multiple imputation model with chained equations was performed based on variables related to sever malaria, obesity status, and missing BMI”. However, we could not find similar information for diabetes (exposure of interest).
2. Khuu et al reported that multiple imputation was not possible because of limited computational power: “Imputation of missing variables was not feasible due to the large size of the NIS and limited computing power”. We still considered this as “no information” because there were no further details about proportion of missing observations.

 

**Q8: Considering that only three articles were reviewed, it would be appropriate to provide a more thorough analysis concerning the diverging results of the two studies assessing risk factors for severe malaria. Any suggestions to why these studies showed different results? Both reported 10% diabetes among severe cases, however a lower proportion of diabetics among non-severe cases in the study by Wyss et al. The authors should consider if the difference could be associated with the selection of malaria cases and definition of severe malaria.**

A8: As pinpointed before, we have discussed in the ‘limitations’ section that a potential driver for the different results between these studies could be the definitions of severe malaria.

**Q9: Implications for research: Concerning generalizability, it is important to mention that the studies were done in different types of populations; while the cross-sectional studies were carried out in a non-endemic setting the case-control study was performed in a malaria endemic area where asymptomatic infections are common and severe manifestations of malaria seldom seen among immunocompetent, non-pregnant adults. Different study designs are required depending on setting and which research question one intends to answer, which also should be pointed out in the discussion. Using a general population sample of diabetics or a population-based cohort study as the authors suggested would not be a reasonable approach in a non-endemic setting where the incidence of malaria is extremely low. In an endemic setting the challenge is instead lack of reliable health and recording systems as well as difficulty in the diagnostics of clinical malaria due to both frequent reinfections and asymptomatic parasitaemia.**

A9: As suggested, in the ‘implications for research’ section we have included a few lines highlighting the differences and strengths of study designs. We agree that a population-based cohort in a non-endemic area would not be reasonable. However, experience has shown us that population-based cohorts in endemic settings where resources are not abundant as in high-income countries, are feasible and provide strong local evidence.

**Q10: Epidemiological context: As the authors emphasize, the prevalence of diabetes is increasing in many countries with high malaria transmission. However, I would suggest the authors to be a bit more careful in drawing conclusions concerning malaria mortality based on country estimates of diabetes and malaria prevalence.**

A10: We have toned down these lines to be more careful in drawing conclusions concerning malaria mortality based on country estimates.

**Q11: Pathways: The possible association of diabetes and risk of malaria infection, and risk of severe disease in an ongoing infection, implies completely different pathobiological mechanisms, which the authors should consider elaborating more on. As an example, there are several studies demonstrating that glucose levels affect parasite growth in vitro, which could have implications for malaria severity. Concerning the hypothesis that metformin use could attract mosquitos it would be reasonable to mention that the same drug also has antimalarial effect (Jones et al 2002).**

A11: We would rather not further elaborate on the mechanisms behind the associations of interest. This work intended to give an epidemiological overview, instead of a comprehensive pathophysiological explanation. Concerning the hypothesis that metformin could attract mosquitos, we have now mentioned the antimalarial effects and included the suggested reference.

**Q12: Again, important to clarify that there were two different objectives in this review, and the included studies analysed two distinct outcomes, thus it is not justified to simply conclude that there is an association between diabetes and malaria.**

A12: As we did in the abstract and introduction, we have also clarified in the conclusion that there were two aims (diabetes-malaria and diabetes-malaria severity).
